# The *Arabidopsis* NMD Factor UPF3 Is Feedback-Regulated at Multiple Levels and Plays a Role in Plant Response to Salt Stress

**DOI:** 10.3389/fpls.2016.01376

**Published:** 2016-09-29

**Authors:** Karina Vexler, Miryam A. Cymerman, Irina Berezin, Adi Fridman, Linoy Golani, Michal Lasnoy, Helen Saul, Orit Shaul

**Affiliations:** The Mina and Everard Goodman Faculty of Life Sciences, Bar-Ilan UniversityRamat-Gan, Israel

**Keywords:** IME, intron-mediated enhancement, NMD regulation, NaCl, negative feedback loop, nonsense-mediated mRNA decay, RNA stability, salt stress

## Abstract

Nonsense-mediated mRNA decay (NMD) is a eukaryotic RNA surveillance mechanism that degrades aberrant transcripts and controls the levels of many normal mRNAs. It was shown that balanced expression of the NMD factor UPF3 is essential for the maintenance of proper NMD homeostasis in *Arabidopsis*. *UPF3* expression is controlled by a negative feedback loop that exposes *UPF3* transcript to NMD. It was shown that the long 3′ untranslated region (3′ UTR) of *UPF3* exposes its transcript to NMD. Long 3′ UTRs that subject their transcripts to NMD were identified in several eukaryotic NMD factors. Interestingly, we show here that a construct that contains all the regulatory regions of the *UPF3* gene except this long 3′ UTR is also feedback-regulated by NMD. This indicates that *UPF3* expression is feedback-regulated at multiple levels. *UPF3* is constitutively expressed in different plant tissues, and its expression is equal in leaves of plants of different ages. This finding is in agreement with the possibility that UPF3 is ubiquitously operative in the *Arabidopsis* NMD pathway. Expression mediated by the regulatory regions of *UPF3* is significantly induced by salt stress. We found that both a deficiency and a strong excess of *UPF3* expression are detrimental to plant resistance to salt stress. This indicates that *UPF3* plays a role in plant response to salt stress, and that balanced expression of the *UPF3* gene is essential for coping with this stress.

## Introduction

Nonsense-mediated mRNA decay (NMD) is an RNA surveillance mechanism that functions in all eukaryotes (reviewed by Celik et al., 2015; Fatscher et al., 2015; He and Jacobson, 2015; Shaul, 2015; Smith and Baker, 2015). Early studies on yeast, nematodes, and mammalian cells showed that transcripts harboring premature termination codons (PTCs) are rapidly degraded (Kinniburgh et al., 1982; Leeds et al., 1991; Pulak and Anderson, 1993). The elimination of PTC-containing transcripts by the NMD mechanism prevents the accumulation of truncated, potentially deleterious, proteins. Moreover, NMD controls the levels of many normal transcripts with features that lead to the recognition of their termination codons (TCs) as premature. The NMD mechanism in mammalian cells is briefly illustrated here. Upon reaching a TC, the ribosome binds the eukaryotic release factors eRF1 and eRF3. Normal translation termination and ribosome recycling, which prohibit NMD, depend on the interaction between eRF3 and the poly(A)-binding protein (PABP; Fatscher et al., 2014; Joncourt et al., 2014). When the ribosome terminates translation far upstream to the poly(A) due to a PTC or a long 3′ untranslated region (3′ UTR), an eRF3-PABP interaction is prevented. Consequently, eRF3 interacts with the NMD factor UPF1 to form the SURF complex, which also includes eRF1 and the protein kinase SMG1 (Kashima et al., 2006). The interaction between the SURF complex and the NMD factors UPF2 and UPF3 leads to the activation of SMG1, which phosphorylates UPF1 and leads to NMD activation (Kashima et al., 2006; Arias-Palomo et al., 2011; Melero et al., 2014).

Introns located ≥50–55 nucleotides (nt) downstream of TCs facilitate NMD (Zhang et al., 1998; Singh et al., 2008). The NMD factor UPF3 interacts with the exon-junction complex (EJC) [a complex of proteins deposited on the mRNA 20–24 nt upstream of exon–exon junctions (EEJ)] before export of the mRNA to the cytoplasm (Lykke-Andersen et al., 2000; Kim et al., 2001). If a TC is located ≥50–55 nt upstream of an EEJ, the terminating ribosome does not remove the EJC. The EJC-bound UPF3 then interacts with UPF2, which bridges between UPF3 and UPF1 (Singh et al., 2007), thereby leading to NMD activation. UPF1 can also bind the mRNA in an EJC-independent, 3′ UTR-length-dependent manner (Hogg and Goff, 2010; Shigeoka et al., 2012; Kurosaki and Maquat, 2013; Zund et al., 2013; Fiorini et al., 2015). Thus, although introns ≥50–55 nt downstream of TCs increase NMD efficiency, they are not essential for this process, and NMD can be activated by long 3′ UTRs alone. NMD can also be activated by upstream open reading frames (uORFs), whose TCs can be recognized as premature due to their long distance from the poly(A) tail.

The basic features of mammalian NMD are conserved in plants, although there are certain regulatory and mechanistic differences between plant and mammalian NMD (reviewed by Shaul, 2015). Plant NMD degrades PTC-containing mRNAs (Hori and Watanabe, 2005; Arciga-Reyes et al., 2006; Yoine et al., 2006b), alternatively spliced transcript isoforms (Kalyna et al., 2012; Drechsel et al., 2013), as well as transcripts derived from pseudogenes, transposable elements, potential natural antisense RNAs, and aberrant mRNA-like non-coding RNAs (Kurihara et al., 2009). Plant NMD also controls the levels of many normal mRNAs that have long (≥300–350 nt) 3′ UTRs (Kertesz et al., 2006; Schwartz et al., 2006; Hori and Watanabe, 2007; Kalyna et al., 2012), introns ≥ 50–55 nt downstream of TCs (Kertesz et al., 2006; Hori and Watanabe, 2007; Nyiko et al., 2013), or uORFs (although not all uORFs activate NMD; Nyiko et al., 2009; Saul et al., 2009; Kalyna et al., 2012; Rayson et al., 2012).

Since the NMD pathway controls the fate of many normal and aberrant plant transcripts, understanding how this pathway is regulated can shed more light on the control of gene expression in plants and plant physiology. However, compared to the current knowledge available about the NMD mechanism, much less is known about how this pathway is regulated. We showed that the maintenance of balanced expression of the *Arabidopsis UPF3* gene is very important for the overall regulation of plant NMD (Degtiar et al., 2015). It is, therefore, important to obtain a full understanding of the regulation of *UPF3* expression. We also showed that *UPF3* is controlled by a negative feedback loop that increases its expression when NMD is inhibited, and restricts its expression when NMD functions properly (Saul et al., 2009). The *Arabidopsis UPF3* transcript is sensitive to NMD owing to its long 3′ UTR (Degtiar et al., 2015). The transcripts of several other eukaryotic NMD factors were also shown to be sensitive to NMD owing to long 3′ UTRs or 3′ UTR introns (Mendell et al., 2004; Rehwinkel et al., 2005; Kerenyi et al., 2008; Huang et al., 2011; Yepiskoposyan et al., 2011; Rayson et al., 2012; Nyiko et al., 2013; reviewed by Huang and Wilkinson, 2012; Karam et al., 2013; Shaul, 2015). However, very little is known about other mechanisms that control the expression of eukaryotic NMD factors. In this work, we show that a construct containing the regulatory regions of *UPF3* but lacking its long 3′ UTR, is also feedback-regulated by NMD. This indicates that *UPF3* expression is feedback-regulated at multiple levels.

It was also interesting to determine whether *UPF3* is differentially expressed in certain plant tissues or growth stages. This can shed light on the question of whether *UPF3* is ubiquitously utilized in the NMD of wild type (WT) plants or, alternatively, if there are certain tissues or growth stages in which a *UPF3*-independent branch of the NMD pathway is normally operative. The latter situation is possible, since *Arabidopsis* plants with a null mutation in the *UPF3* gene are viable. Because a complete loss of NMD function in *Arabidopsis* is lethal (Arciga-Reyes et al., 2006; Yoine et al., 2006a), NMD should still be functional in plants with a loss of *UPF3* function [although with a much lower efficiency (Hori and Watanabe, 2005)]. In mammalian cells, there are branches of the NMD pathway that do not require the involvement of all known NMD factors (Gehring et al., 2005).

A link between NMD and plant-stress response was established. Biotic stress inhibits NMD in plants, thereby initiating a signaling cascade that elevates plant defense (Jeong et al., 2011; Rayson et al., 2012; Riehs-Kearnan et al., 2012; Shi et al., 2012; Gloggnitzer et al., 2014; reviewed by Shaul, 2015). It was also reported that the expression of *UPF1* and *UPF3* is downregulated by biotic stress (Jeong et al., 2011).

Compared to the response to biotic stress, much less is known about the correlation between NMD and other types of plant stress responses. It was shown that NMD is inhibited by salt stress (Drechsel et al., 2013), but it is not known whether salt stress affects the expression of NMD factors. To increase our understanding of the correlation between NMD and plant stress responses, we examined the impact of salt stress on *UPF3* expression. We also examined the impact of deficient or excess *UPF3* expression on the response to salt stress.

We show here that *UPF3* is expressed in all plant organs and at all growth stages, in agreement with the possibility that *UPF3* is ubiquitously operative in the *Arabidopsis* NMD pathway. Our finding that a construct including *UPF3* regulatory regions but lacking its long 3′ UTR is also controlled by NMD, indicates that *UPF3* expression is feedback-regulated by NMD at multiple levels. We also show that *UPF3* is significantly induced by salt stress. We found that both a deficiency and a strong excess in *UPF3* expression are detrimental to plant resistance to salt stress. This indicates that *UPF3* plays a role in plant response to salt stress, and that the balanced expression of the *UPF3* gene is essential for coping with this stress.

## Materials and Methods

### Mutant Lines, Plant Transformation, and Expression Analysis

The WT *Arabidopsis thaliana* (L.) plants were of the Col-0 accession, and the mutant plants were homozygous and in the Col-0 background. The selection of homozygous progenies of the *Arabidopsis* T-DNA insertion mutants (Alonso et al., 2003) SALK_112922 (*upf1-5*; Arciga-Reyes et al., 2006) and SALK_025175 (*upf3-1*; Hori and Watanabe, 2005) was described (Saul et al., 2009). The plants were transformed using the floral dip technique and selected on 20 μg/mL hygromycin B. The plants used for expression analysis were germinated on MS (Duchefa Biochemie BV) plates, and grown in climate-controlled growth rooms or greenhouses in a photoperiod of 16 h light and 8 h dark. Growth in hydroponics was carried out as described (Berezin et al., 2013).

### Generation of Constructs

Construct U3::GUS is similar to construct U3::R described in Degtiar et al. (2015), except that the coding sequence of UPF3 was replaced with that of β-glucuronidase (GUS). The U3::GUS construct included the *UPF3* promoter, the *UPF3* 5′ UTR (which includes an uORF), and the *UPF3* terminator (which includes the last intron of this gene). The cloning of these sequence elements, which were similar to those utilized in the U3::R construct, was described (Degtiar et al., 2015). The first intron of *UPF3*, which was present in the coding sequence of *UPF3* in the U3::R construct, was inserted into the GUS coding sequence as described in Supplementary Figure 1. Construct U3::GUS-In was identical to construct U3::GUS except that it did not include the first intron of *UPF3* (but did include all the extra sequences added to the GUS coding sequence to allow the splicing of this intron–see Supplementary Figure 1B). Construct U3::GUS-NOS was identical to U3::GUS except that the native terminator of *UPF3* was replaced with the short nopaline synthase (NOS) terminator. The generation of the U3::NR construct was previously described (Degtiar et al., 2015). All these constructs were cloned into a binary vector that included the coding sequence of *Escherichia coli* hygromycin phosphotransferase, which confers hygromycin B resistance, immobilized into *Agrobacterium tumefaciens* strain *EHA105*, and used for the stable transformation of *Arabidopsis* (Col-0) plants.

### RNA Extraction and Northern Blot Analysis

Total RNA was extracted using the TRI-Reagent (Sigma). RNA samples were denatured with glyoxal (Sigma) and fractionated on 1% agarose gels. Gel preparation and fractionation were carried out with 10 mM NaPi buffer, pH 7.0. The gels were blotted onto a Zeta-Probe GT membrane (Bio-Rad) with 25 mM NaPi buffer, pH 7.0. RNA was fixed by UV. The membranes were stained by 0.02% methylene blue in 0.3 M sodium acetate (pH 5.5) to visualize the ribosomal RNA, and then rinsed in H_2_O. Hybridization was carried out using the DIG-labeling system (Roche Diagnostics GmbH) according to the manufacturer’s instructions. The primers used for probe preparation were: *UPF3*, 5′-AAGGCACCAGAAGATG-3′ and 5′-GGATCCACATTTGCTTCTCAT-3′; *EF1a (AT1G07920)*, 5′-CACGTCGATTCTGGAAAGTC-3′ and 5′-TGATAACACCGACTGCAACAG-3′.

### Quantitative Real-Time PCR and Data Analysis

Total RNA was extracted using the TRI-reagent (Sigma). RNA concentration was quantified using a spectrophotometric device, and RNA quality was checked on gel. An amount of 2 μg of each RNA sample was DNase-treated using the TURBO DNA-free Kit (Ambion). Non-reverse transcription control (NRT) samples were amplified by PCR to verify the absence of residual DNA. The DNase-treated RNA samples were then used for reverse transcription using the RevertAid First Strand cDNA Synthesis Kit (Thermo Scientific). Quantitative RT-PCR was performed on a Bio-Rad CFX96 Real-Time PCR system (Bio-Rad Laboratories) using the SuperReal PreMix plus SYBR Green RT-PCR kit (Tiangen). Primer efficiency calculations and data analyses were carried out using the Bio-Rad CFX Manager software v3.1. The ‘no template controls’ contained water instead of cDNA. Primer efficiencies were determined using a fivefold dilution series of a representative cDNA sample measured in triplicate. The efficiencies of all primer pairs were 100 ± 10%. Expression levels of *UPF3* in each plant type were calculated following analysis of four biological replicates, with three technical replicates for each sample, using the standard curve (which takes into account primer efficiency). *Arabidopsis YLS8* gene (AT5G08290) was used as an internal standard (Czechowski et al., 2005), using the primers indicated in Czechowski et al. (2005), to normalize the variations of cDNA concentrations. This control gene showed very similar transcript levels among all samples. The thermal cycling program was 95°C for 15 min, followed by 40 cycles of 95°C for 10 s and 60°C for 30 s. Primer sequences were: *UPF3*, 5′-AAGAAGAGGTGGTGATTGG-3′ and 5′-GGATCCACATTTGCTTCTCA-3′; *YLS8*, 5′-TTACTGTTTCGGTTGTTCTCCATTT-3′ and 5′-CACTGAATCATGTTCGAAGCAAGT-3′.

### Quantitative GUS Analysis

Quantitative measurement of GUS activity was carried out using the fluorometric assay (Breyne et al., 1993). Plant material was ground in liquid nitrogen and extracted in a buffer containing 100 mM NaPO_4_, pH 7, 10 mM Na_2_EDTA, pH 8, 10 mM β-mercaptoethanol, and 0.1% (v/v) Triton X-100. Following centrifugation (5 min, 14,000 ×*g*, 4°C), the supernatant was collected and the concentration of proteins was determined using the Bradford reagent (Sigma). Samples including equal amounts of protein were suspended in 250 μl extraction buffer including 1.2 mM (final concentration) of the fluorescent GUS substrate 4-methylumbelliferyl-β-D-glucuronide (MUG; Duchefa Biochemie BV). GUS activity was assayed on a 96-well fluorescent plate reader (Fluoroscan II, Lab Systems) with the excitation wavelength set at 350 nm and the emission wavelength at 460 nm. GUS activity (milli units⋅mg protein^-1^) was calculated from the slope of the line generated from measures taken at 3-min intervals during 1 h, with respect to the slope of commercial, pure GUS enzyme (Roche Diagnostics GmbH).

### Histochemical GUS Analysis

For histochemical analysis of GUS activity, plants were incubated for 1–2 days at 37°C in a medium that included 50 mM NaPO_4_, pH 7.2, 0.5 mM K_3_Fe(CN)_6_, 20% (v/v) methanol, and 1 mM X-Gluc A (Duchefa Biochemie BV). This solution was vacuum-infiltrated into the plants. Stained plants were cleared in 70% (v/v) ethanol. Photographs were taken with an Olympus AH3-RFCA microscope or with an Olympus SZX-12 binocular.

## Results

### The First Intron of *UPF3* Is Essential for its Expression

**FIGURE 1:**
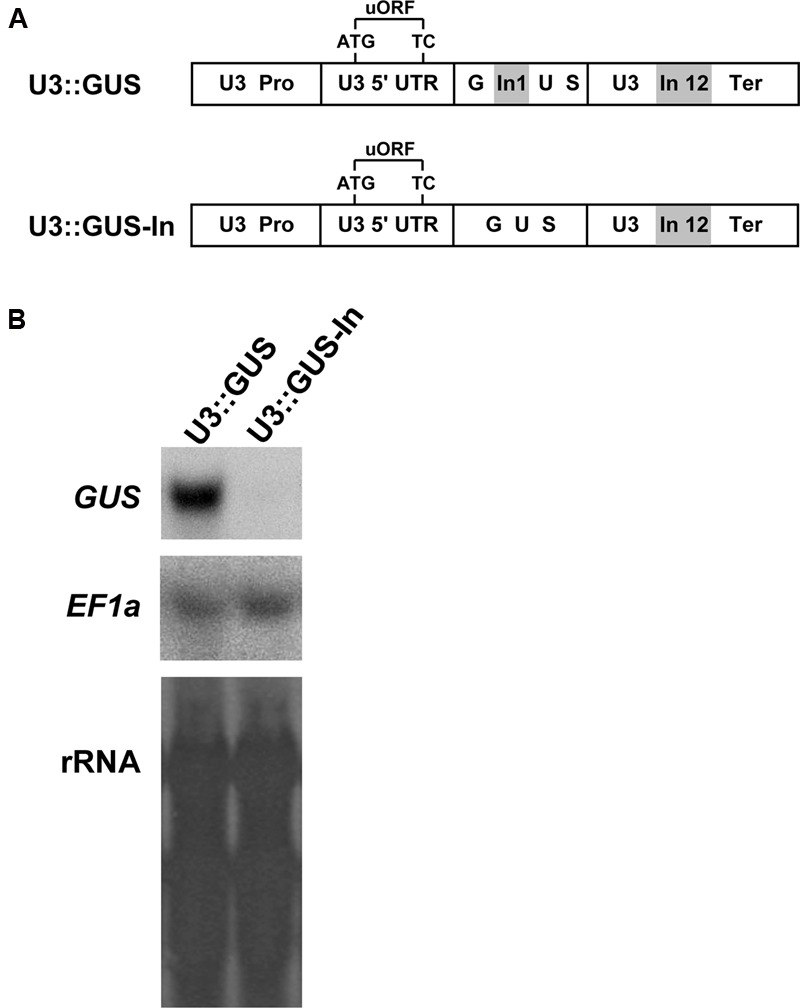
**The first intron of *UPF3* is essential for its expression. (A)** The constructs used for expression analysis. The U3::GUS construct included the *UPF3* promoter (U3 Pro), the 5′ UTR of *UPF3* (U3 5′ UTR), which contains an upstream open reading frames (uORF), the coding sequence of β-glucuronidase (GUS), which includes the first intron of *UPF3* (indicated by the letters In1 on gray background), and the terminator of *UPF3*, which includes the 12th intron of this gene (indicated by the letters In12 on gray background). The terms ATG and TC refer to the initiation and termination codons, respectively, of the uORF. The fusion between GUS and the first intron is illustrated in Supplementary Figure 1B. The U3::GUS-In construct was identical to the U3::GUS construct, except that it did not include the first intron of *UPF3*. **(B)** Northern blot hybridization of RNA extracted from *Arabidopsis* (Col-0) plants expressing the constructs illustrated in **(A)** with the *GUS* or *EF1a* gene probes. The figure shows a representative pair from the 20 biological replicates that were hybridized for each construct. For each construct, RNA was extracted from 2-week-old plants grown in 20 plates (which constituted the 20 biological replicates). The 20 plates of each construct included a total of ∼1000 plants. These 1000 plants were germinated from mixtures including ∼40 T2 progeny plants from each of the 25 independent T1 transformants regenerated for each construct. The corresponding methylene blue staining of the ribosomal RNA (rRNA) is shown below the blots.

Proper balancing of *UPF3* expression is crucial for NMD homeostasis in *Arabidopsis* (Degtiar et al., 2015). We thus wanted to obtain a full understanding of the regulation of *UPF3* expression. For this, we created the U3::GUS construct, in which the reporter GUS was fused to all the regulatory elements of the *UPF3* gene (**Figure 1A**). These elements included the native promoter, 5′ UTR, terminator, and the first and last introns of this gene. The last (12th) intron of *UPF3* is located in its 3′ UTR. However, we found that this intron, which is located less than 50 nt downstream of the TC, did not affect *UPF3* expression (Degtiar et al., 2015). The first intron of *UPF3* was also included in the U3::GUS construct because some introns, particularly first introns, can play important roles in gene expression through the intron-mediated enhancement (IME) mechanism [reviewed by Gallegos and Rose (2015)]. However, many introns are incapable of affecting expression. The first intron of *UPF3* is considerably longer than other introns of this gene (Supplementary Figure 1A). To determine if this intron plays a role in *UPF3* expression, we created the U3::GUS-In (-In stands for minus intron) construct, which did not include this intron (**Figure 1A**).

In the U3::GUS construct, the first intron was inserted into the GUS coding sequence flanked by several nucleotides of the original coding sequence of *UPF3*, to ensure the proper splicing of this intron (Supplementary Figure 1B). The reading frame of these few nucleotides was altered (Supplementary Figure 1B), to prevent the translation of the first amino acids of *UPF3* and a consequent putative dominant negative effect. In the U3::GUS-In construct, GUS was fused to essentially the same sequences, including the few flanking nucleotides, but excluding the intron. The two constructs were stably transformed into *Arabidopsis*, and GUS transcript level was determined (**Figure 1B**). GUS transcript in plants expressing the construct that lacked the intron was below the detection limit. The *EF1a* mRNA was used as a control to demonstrate that this did not result from mRNA degradation in the U3::GUS-In plants. This indicated that the first intron of *UPF3* is important for expression of this gene. To ensure reliable data, expression analysis was carried out in plants expressing the U3::GUS construct, which included this intron.

### *UPF3* Is Ubiquitously and Stably Expressed in Different Plant Organs and Growth Stages

**FIGURE 2:**
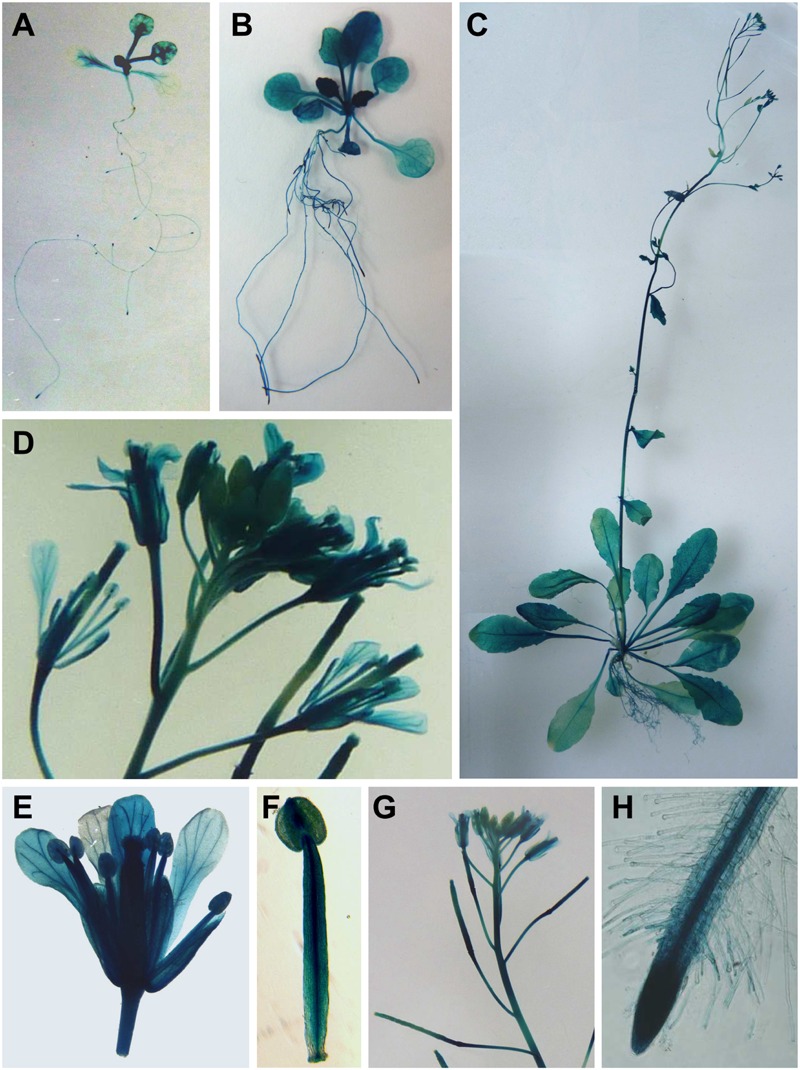
**Histochemical GUS staining of plants expressing the U3::GUS construct. (A)** A 14-day-old plant. **(B)** A hydroponically grown 25-day-old plant. **(C)** A soil-grown plant at the reproductive stage. **(D)** An inflorescence. **(E)** A flower. **(F)** A stamen. **(G)** Inflorescence and siliques. **(H)** A root tip.

Young seedlings of transgenic plants expressing the U3::GUS construct showed relatively uniform GUS staining in true leaves and in roots (**Figures 2A,H**). GUS staining in the cotyledons was lower than in true leaves. Staining was observed in all parts of 25-day-old plants, including roots and leaves (**Figure 2B**). Expression was also observed in all parts of plants at the reproductive stage, including roots, rosette and cauline leaves, inflorescence stems, flowers, and siliques (**Figures 2C–G**). Thus, unless UPF3 has other, yet-unidentified functions, this expression pattern is in agreement with the possibility that the NMD process is functional in all plant parts and at all developmental stages, and that UPF3 is ubiquitously utilized in the NMD pathway of WT *Arabidopsis* plants.

**FIGURE 3:**
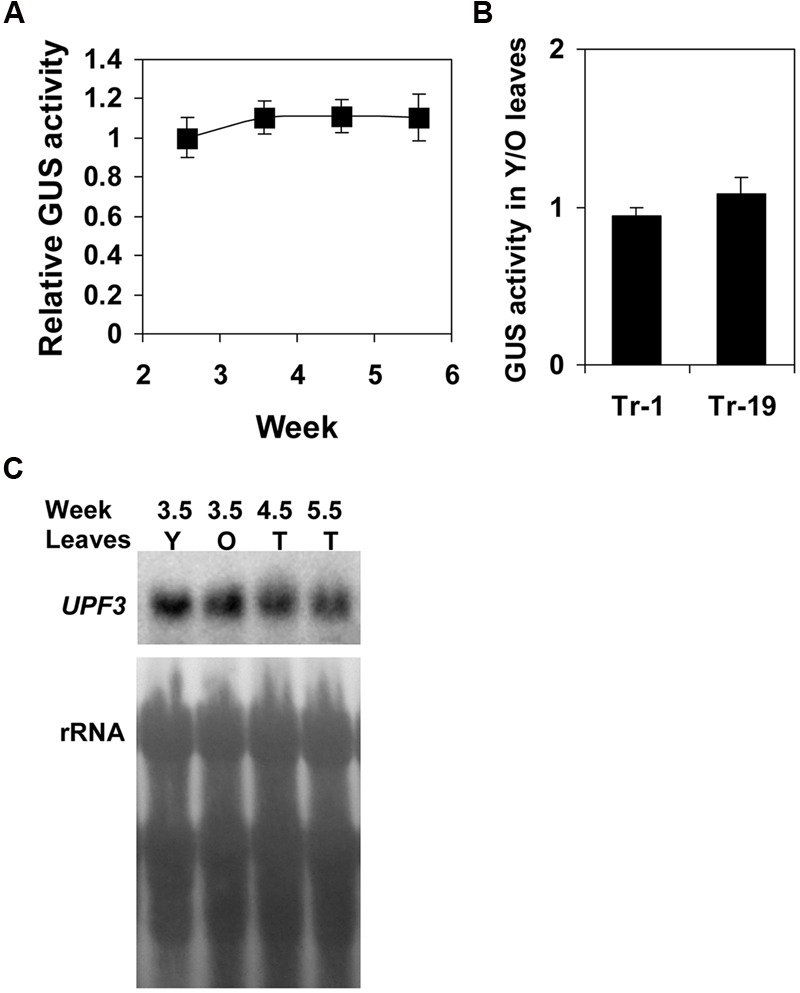
***UPF3* expression is equal in leaves at different growth stages. (A)** GUS activity in rosette leaves of homozygous transformed plants expressing the U3::GUS construct. Seven biological replicates, each including the leaves of four plants, were analyzed at each age. The plants were germinated at different times and, subsequently, all plants were harvested at the same time to minimize environmental variation. The graph shows the mean of GUS activity of plants at different ages, relative to the activity of 2.5-week-old plants. Error bars represent the standard error (SE). **(B)** GUS activity in younger relative to older leaves of two different homozygous transformed plants (named Tr-1 and Tr-19) expressing the U3::GUS construct. Seven biological replicates were analyzed for each plant. Each replicate was composed of five 25-day-old plants grown in the greenhouse. The older leaves were true leaves numbers 1–4, and the younger leaves were true leaves numbers 5–7. All the younger and older leaves of each group of five plants were pooled and analyzed, and the ratio of GUS activity (GUS units per mg protein) of the younger relative to the older leaves (Y/O) was determined. Each column shows the mean of the Y/O values of the seven biological replicates analyzed for each plant type. Error bars represent the SE. **(C)** Northern blot hybridization with the *UPF3* gene probe of wild type (WT) plants at the indicated ages. For 3.5-week- (25-day-) old plants, the younger (Y) and older (O) rosette leaves were harvested separately while, for the other ages, the total (T) rosette leaves were harvested together. Plant growth and harvest, as well as the separation to different samples, were carried out as described in **(A,B)**.

Although GUS staining was observed in plants at different ages, quantitative GUS analysis was necessary to determine if there were differences in the level of expression at different growth stages. It was found that the expression in rosette leaves of plants at different growth stages was equal (**Figure 3A**). Expression was also equal in younger and older leaves of the same plant (**Figure 3B**). The results obtained using the reporter gene were supported by analysis of *UPF3* mRNA levels (**Figure 3C**). *UPF3* transcript levels were similar in younger and older leaves of 25-day-old plants, and were also similar in the leaves of older plants. Altogether, these data indicated that *UPF3* expression in leaves is stable throughout plant growth.

### The Induction of *UPF3* under NMD Inhibition Depends Not Only on Its Long 3′ UTR but Also on Other Regulatory Elements of This Gene

**FIGURE 4:**
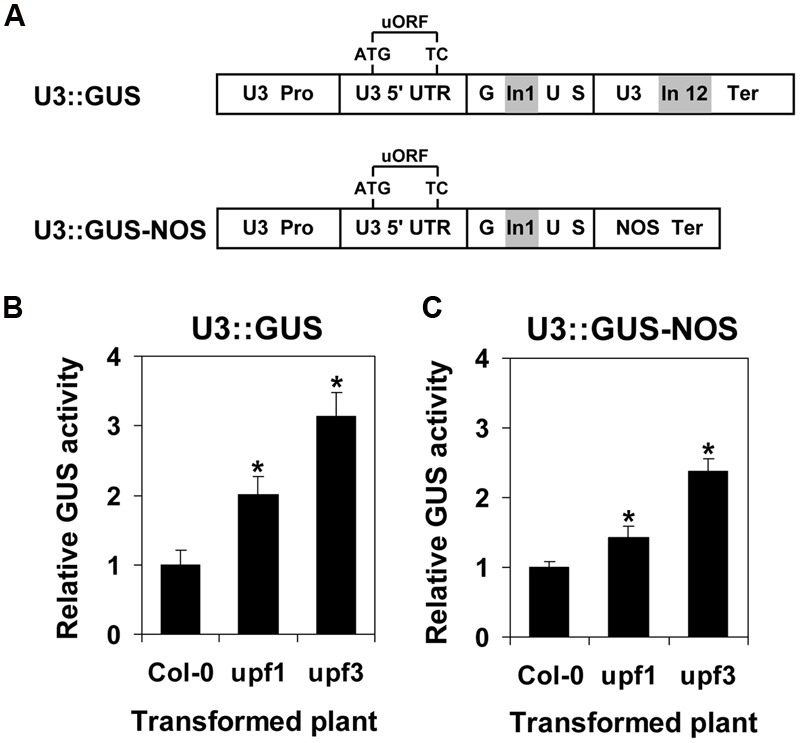
**Expression of the U3::GUS and U3::GUS-NOS constructs in transformed WT (Col-0) plants compared with transformed *upf1-5* or *upf3-1* mutants. (A)** The constructs expressed in the plants. The U3::GUS construct was described in the legend of **Figure 1**. Construct U3::GUS-NOS was identical to U3::GUS except that the *UPF3* terminator was replaced with the short NOS terminator. **(B,C)** GUS activity of Col-0 plants, as well as *upf1-5* and *upf3-1* mutants, transformed with the U3::GUS **(B)**, or U3::GUS-NOS **(C)** constructs. Each column shows the mean of GUS activity (normalized to the activity of transformed Col-0 plants) of 15 biological replicates obtained in two experiments. Each biological replicate was composed of ∼50 2-week-old plants grown on the same MS plate (thus, each column represents the average GUS activity of ∼750 plants). These 750 plants were composed of ∼19 T2 progeny plants from each of the ∼40 independent T1 transformants that were regenerated for each construct in each plant. Error bars represent the SE. An asterisk indicates a statistically significant difference (*p* < 0.05) between the transformed NMD mutants and WT (Col-0) plants, as determined by Student’s *t*-test.

We previously showed that the long 3′ UTR of *UPF3* subjects the transcript of this gene to NMD (Degtiar et al., 2015). It was interesting to learn whether, besides the long 3′ UTR, other regulatory elements of the *UPF3* gene can also confer NMD sensitivity. To address this question, we compared the expression in WT and NMD mutant plants of the U3::GUS construct, which contained all the regulatory regions of *UPF3*, including the long (545 nt) 3′ UTR, with that of the U3::GUS-NOS construct, which was identical except having the short (∼180 nt long; Bevan et al., 1983) NOS 3′ UTR (**Figure 4A**). The NMD mutant plants utilized were *upf1-5* and *upf3-1*. The *upf1-5* mutants have severely reduced, but not completely eliminated expression of *UPF1* (a null mutation in *UPF1* is lethal in *Arabidopsis*; Arciga-Reyes et al., 2006; Yoine et al., 2006a). The *upf3-1* mutants do not have intact *UPF3* mRNA (Hori and Watanabe, 2005).

As expected, the expression of the U3::GUS construct was higher in stably transformed NMD mutants as compared with transformed WT (Col-0) plants (**Figure 4B**). This can be explained by the presence in the U3::GUS construct of the long 3′ UTR of *UPF3*, which renders it NMD-sensitive (Degtiar et al., 2015). The higher expression of the U3::GUS construct in *upf3-1* compared with *upf1-5* mutants is in accord with the indication that, in terms of NMD impairment, the *upf3-1* allele is stronger than the *upf1-5* allele (Kalyna et al., 2012). Interestingly, increased expression in transformed NMD mutants compared with transformed WT plants was also observed for the U3::GUS-NOS construct, which did not include the long 3′ UTR of *UPF3* (**Figure 4C**). This indicated that besides the long 3′ UTR, other regulatory elements of the *UPF3* gene can also confer NMD sensitivity. We previously showed that the uORF of *UPF3* does not expose it to NMD (Degtiar et al., 2015). It is, thus, possible that the *UPF3* promoter includes NMD responsive elements. The extent of induction by NMD inhibition was higher for plants expressing the U3::GUS as compared with the U3::GUS-NOS construct (**Figures 4B,C**). This indicates that the impacts of the long 3′ UTR and the other regulatory elements that confer NMD responsiveness to the *UPF3* gene are cooperative.

### The Regulatory Regions of *UPF3* Are Induced by Salt Stress

It was shown that NMD plays a role in plant response to biotic stress, and the expression of *UPF1* and *UPF3* is affected by biotic stress (Jeong et al., 2011; Rayson et al., 2012; Riehs-Kearnan et al., 2012; Shi et al., 2012; Gloggnitzer et al., 2014; reviewed by Shaul, 2015). Here, we investigated whether salt stress affects the expression of *UPF3* regulatory regions. For this, plants expressing the U3::GUS and U3::GUS-NOS constructs were grown on plates including 100 mM NaCl. We compared the impact of NaCl, which imposes both salt and osmotic stress, with that of sorbitol, which imposes osmotic stress only. The sorbitol concentrations used were 200 mM (providing equal osmolarity to 100 mM NaCl) and 250 mM (providing increased osmolarity).

**FIGURE 5:**
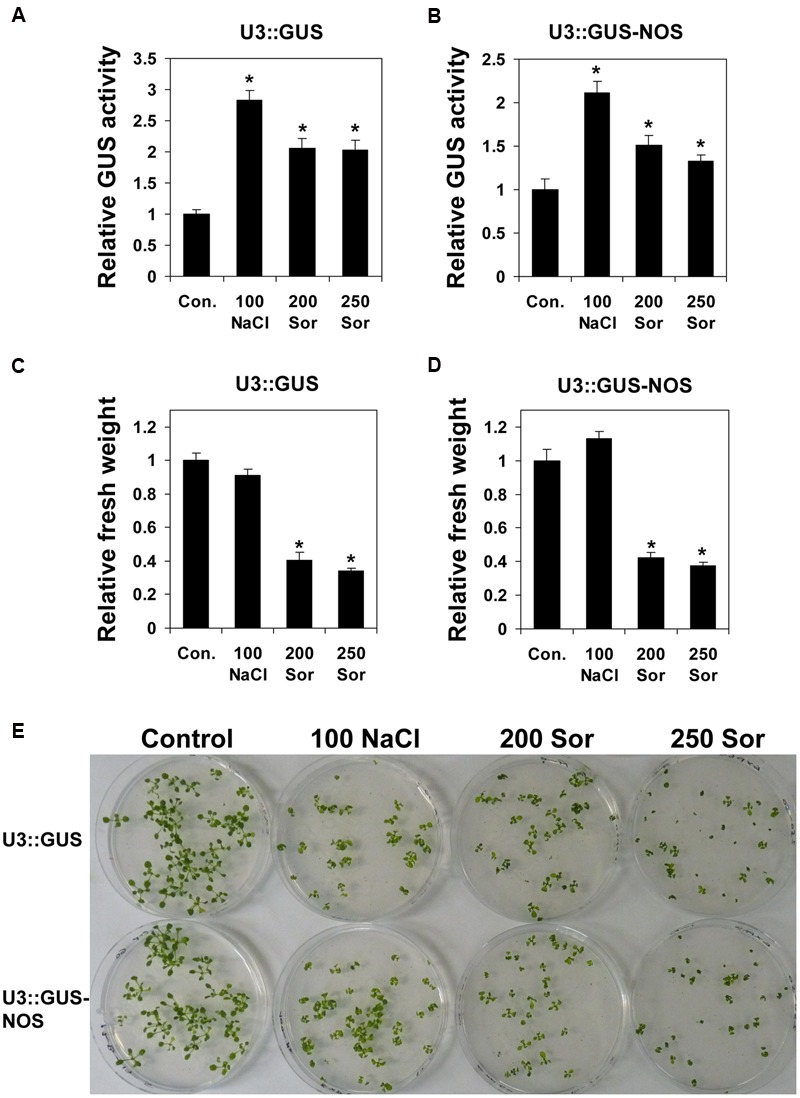
**Induction of *UPF3* regulatory regions by salt stress. (A,B)** WT (Col-0) plants expressing the U3::GUS **(A)** or U3::GUS-NOS **(B)** constructs were germinated on MS plates (control), or on plates containing 100 mM NaCl, 200 mM sorbitol, or 250 mM sorbitol. For each construct, we utilized homozygous transformed plants expressing a single copy of the transgene. Each column represents the mean GUS activity of 10 biological replicates. Each biological replicate was composed of ∼50 2-week-old plants grown on the same plate (thus, each column represents the average GUS activity of ∼500 plants). For each construct, data were normalized to the GUS activity of the control treatment. **(C,D)** The fresh weight of plants expressing the U3::GUS **(C)** or U3::GUS-NOS **(D)** constructs following the different treatments. Each column represents the mean fresh weight of 10 biological replicates. Each replicate was the average fresh weight of a single plant determined according to the weight and number of plants grown on a single plate (thus, each column shows the average weight of about 500 plants grown in 10 plates). All data were normalized to the control treatments. Error bars represent the SE. An asterisk indicates a significant difference (*p* < 0.05) between the control and the NaCl or sorbitol treatments as determined by Student’s *t*-test. **(E)** Representative plates at the time of harvesting.

As exhibited by plants expressing both constructs, salt treatment induced the expression mediated by the regulatory regions of *UPF3* (**Figures 5A,B**). Increased expression, although to a lower extent, was also observed upon treatment with sorbitol (**Figures 5A,B**). We attempted to estimate the extent of growth inhibition mediated by these stress treatments (NaCl and sorbitol) by measuring the plants’ fresh weight (**Figures 5C,D**). Although plants exposed to salt stress exhibited clear stress symptoms (**Figure 5E**), their fresh weight was not reduced compared with plants grown on the control medium (**Figures 5C,D**). This may result from water accumulation in the salt-grown plants, whose leaves had a succulent appearance and were apparently thicker than leaves of plants grown on the other media (control or sorbitol). Nevertheless, it was possible to conclude that induction in the presence of NaCl did not result from the mere impairment of plant growth, because induction in the presence of 250 mM sorbitol, which inhibited growth to a larger extent than did 100 mM NaCl (**Figure 5E**), was not greater than that mediated by 100 mM NaCl.

### Balanced Expression of *UPF3* Is Essential for Plant Resistance to Salt Stress

**FIGURE 6:**
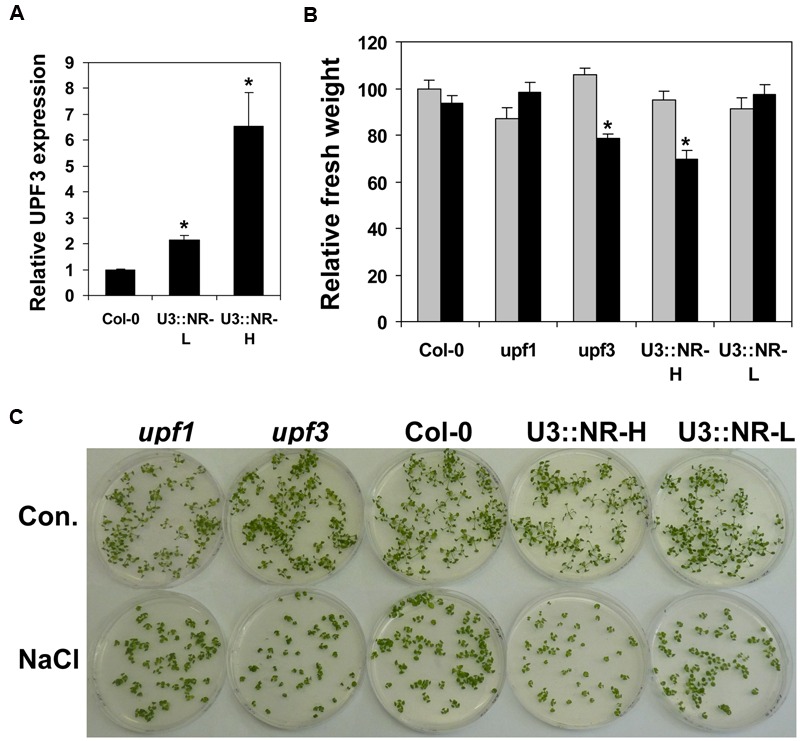
**Plants with deficient or highly excessive *UPF3* expression exhibit impaired salt tolerance.** In order to compare salt tolerance, WT (Col-0) plants, *upf1-5* and *upf3-1* mutants, as well as plants overexpressing UPF3 under the control of its own regulatory sequences (U3::NR-L and U3::NR-H), were germinated in the absence or presence of NaCl. **(A)** qRT-PCR analysis showing the extent of *UPF3* overexpression in each of the two homozygous transformed plants (U3::NR-L and U3::NR-H) used in this experiment. Samples from four biological replicates (four different plates including ∼50 plants each), each analyzed using three technical replicates, were used for determination of *UPF3* transcript content in each plant type, and data were normalized to *UPF3* expression in WT plants. Error bars represent the SE. An asterisk indicates a significant difference (*p* < 0.05) between the WT and the transformed plant, as determined by Student’s *t*-test. **(B)** Each column represents the mean fresh weight of 10 biological replicates grown on the control medium (gray columns) or on plates containing 100 mM NaCl (black columns). Each replicate was the average fresh weight of a single plant determined according to the weight and number of 2-week-old plants grown in a single plate (thus, each column shows the average weight of about 500 plants grown in 10 plates). It was verified that all seeds germinated at the same time. All data were normalized to the control treatment of WT plants. Error bars represent the SE. An asterisk indicates a highly significant difference (*p* < 0.001) between the control and the NaCl treatment of the same plant genotype, as determined by Student’s *t*-test. **(C)** Representative plates at the time of harvesting.

We investigated whether the level of *UPF3* expression affects plant response to salt stress. For this, we utilized WT (Col-0) plants, *upf3* (and also *upf1*) mutants, and plants with increased expression of *UPF3*. WT plants with increased expression of *UPF3* (named U3::NR plants) were obtained in a previous study (Degtiar et al., 2015). These plants, in which the coding sequence of *UPF3* was expressed under the control of its own promoter and the NOS terminator, showed an increase in *UPF3* expression compared with WT plants. We created two types of homozygous plants (with a single copy of the transgene), which had either an about twofold increase in *UPF3* expression, or about a sixfold increase in *UPF3* expression. These plants were named U3::NR-L (L stands for low) and U3::NR-H (H stands for high), respectively. This level of expression was further verified (**Figure 6A**) in plants grown during the experiment presented in **Figure 6B**.

The WT (Col-0) plants, NMD mutants, and U3::NR plants were grown in control or NaCl including media, and their fresh weight was determined. We verified that all plants germinated at the same time. Similar to the findings presented in **Figures 5C,D**, the fresh weight of WT (Col-0) plants grown in 100 mM NaCl was similar to that of plants grown in the control medium (**Figure 6B**). A similar observation was made for *upf1* mutants and the U3::NR-L plants, which had a twofold increase in *UPF3* expression (**Figures 6A,B**). Interestingly, both *upf3* mutants and plants with about a sixfold increase in *UPF3* expression exhibited a significant reduction in their fresh weight under salt stress compared with plants grown in the control medium (**Figure 6B**). These findings are reflected by the phenotype of these plants (**Figure 6C**). U3::NR-H plants and *upf3* mutants grown on the control medium did not show reduced fresh weight compared with WT plants (**Figure 6B**). To conclude, plants with either deficient or highly excessive *UPF3* expression exhibit impaired salt tolerance. This indicates that balanced expression of *UPF3* is essential for plant resistance to salt stress (see Discussion for a detailed explanation of this conclusion).

## Discussion

Very little is known about NMD regulation in plants and the mechanisms controlling the expression of plant NMD factors. The proper regulation of *UPF3* expression is crucial for NMD homeostasis in *Arabidopsis* (Degtiar et al., 2015). It is, therefore, interesting to obtain a full understanding of the mechanisms that regulate *UPF3* expression. To address this topic, we utilized a reporter construct that included all the regulatory regions of the *UPF3* gene. We first showed that the first intron of *UPF3* is essential for the expression of this gene. Some introns, particularly first introns, are capable of enhancing gene expression in a process termed IME (reviewed by Gallegos and Rose, 2015). While in some cases no expression is seen in the absence of introns, many introns do not affect gene expression. Whereas no expression could be observed in the absence of the first intron of *UPF3*, the last intron located in the 3′ UTR of *UPF3* was unable to restore the enhancing function of the first intron (**Figure 1**). Due to its essential function, the first intron of *UPF3* was included in all reporter gene constructs utilized in this study. This increased the reliability of the data obtained.

*UPF3* showed rather uniform expression in different plant tissues. Although some proteins involved in the NMD process are also engaged in other functions (Isken and Maquat, 2008), NMD-unrelated functions have not yet been assigned to UPF3. Thus, unless such functions are identified, the expression pattern observed here is in agreement with the possibility that UPF3 is ubiquitously utilized in the NMD of WT plants. Expression was similar in both the young and mature leaves of the same plant, and was also stable in plants at different growth stages. This suggests that the NMD process is functional at all growth stages.

In both plant and mammalian cells, the feedback loops controlling the expression of NMD factors have been attributed only to long 3′ UTRs, 3′ UTR introns, and/or uORFs (see Introduction). We previously showed that the long 3′ UTR of *UPF3* exposes this gene to NMD (Degtiar et al., 2015). Interestingly, we show here that a construct that contains all the regulatory regions of the *UPF3* gene except this long 3′ UTR is also feedback-regulated by NMD. Since the uORF of *UPF3* does not expose its transcript to NMD (Degtiar et al., 2015), it is possible that this additional layer of NMD sensitivity results from elements in the *UPF3* promoter. Our findings revealed that *UPF3* is feedback-regulated at multiple levels. However, the effects of the long 3′ UTR and the other element(s) conferring NMD responsiveness were cooperative, and regulation of *UPF3* by its long 3′ UTR was essential for the maintenance of NMD homeostasis in *Arabidopsis* (Degtiar et al., 2015).

Interestingly, the expression of the U3::GUS and U3::GUS-NOS constructs was induced by salt (**Figures 5A,B**). Salt excess imposes two major adverse effects on plants: osmotic stress and ion (Na^+^ and Cl^-^) toxicity (Greenway and Munns, 1980). The induction of U3::GUS and U3::GUS-NOS expression was stronger in the presence of NaCl than upon treatment with sorbitol at equivalent or even higher osmolarity. This indicates that the induction of expression under salt stress results from both the osmotic and the ion toxicity components of the stress.

Our finding that *upf3* mutants have increased sensitivity to salt stress as compared to the WT plants points to a role of UPF3 in plant response to salt stress. The increased salt sensitivity of *upf3* mutants could be a secondary impact related to the role of UPF3 in NMD management. According to this suggestion, NMD impairment alters the expression of genes that are essential for plant response to salt stress. The finding that *upf1* mutants are less sensitive to salt stress than *upf3* mutants (**Figure 6**) may be explained by the observation that the *upf3-1* allele is stronger than the *upf1-5* allele in terms of NMD impairment (Kalyna et al., 2012).

It was shown that salt stress inhibits NMD (Drechsel et al., 2013). It is possible that the moderate increase in the expression of *UPF3* during salt stress is helpful for coping with this inhibition. It was, therefore, interesting to determine the impact of increased expression of *UPF3* on plant response to salt stress. However, plants with a twofold increase in *UPF3* expression (the U3::NR-L plants) did not show increased salt tolerance as compared with the WT plants (**Figure 6**). It is reasonable that increased expression of *UPF3* is just one of several mechanisms utilized by plants to cope with salt stress, but alone, it is not sufficient for eliminating this stress. Interestingly, plants with a strong increase in *UPF3* expression (the U3::NR-H plants) showed, similar to the *upf3* mutants, reduced salt tolerance compared with the WT plants (**Figure 6**). This finding can be explained by our previous observation that not only a deficiency but also an excess of UPF3 can impair NMD (Degtiar et al., 2015).

Altogether, our data show that both deficiency and a strong excess in *UPF3* expression are detrimental to the plant resistance to salt stress. This indicates that *UPF3* plays a role in the plant response to salt stress, and that a balanced expression of the *UPF3* gene (avoiding both a deficiency and a strong excess) is essential for coping with this stress. Thus, the feedback regulation of *UPF3* expression, which prevents the imbalanced expression of this gene, is essential for plant salt response.

## Author Contributions

KV, MAC, IB, AF, LG, ML, and HS performed the experiments and participated in data analysis. OS designed the experiments, participated in data analysis, and wrote the manuscript.

## Conflict of Interest Statement

The authors declare that the research was conducted in the absence of any commercial or financial relationships that could be construed as a potential conflict of interest.
