# Supplementary material for: The Arabidopsis NMD Factor UPF3 Is Feedback-Regulated at Multiple Levels and Plays a Role in Plant Response to Salt Stress
Source: Front Plant Sci. 2016 Sep 29;7:1376. doi: 10.3389/fpls.2016.01376 (PMC5040709; doi:10.3389/fpls.2016.01376)
Supplement: Supplementary file 1 [file Image_1.PDF]

Supplementary Data

Supplementary Figure 1

A

aacaccaaaaacagaacaaaacagagccagaagaatcaaacctaaccacagaatcaacagatgaataagcaatca **AT1G33970**
agtaaagaagatagagacgaaagaagattacttgcttaaaaaccggagaagctaaagcgacaatcttagagaaaa
atatctttttggactttatacagaatcagtaaaacctgacaagaccagaagtctctgcgaaacaaactcgtact
ttatcacctgcgacgcggatcacctattagattgattatacaccgtacgatgtattattataatcttcatcctac
ggtaagcccaataacaagcccatttcacaaactatcagagtaattatctgccgtccgatgccttaaaacttaatc
cgacggttgaaacatataattaaggcccattagaggcccatataggttaaaaagtccacgtagaccgaagattga
tttgtctacgggtattttaatttttttcttttcccagaggaaagggaagagaggaaacaaaaattagggcaaaatt **AT1G33980.1**
agcgtattgatgtgtttcacttttcttcttcttcttcttcatcgtgttcgtgtttgatttgcgactttctccaag
gaaaatttagggttttcg**ATG**AAGGAACCTTTGCAGAAGAAGAAGGTGGTTGTTCGTCACTTGCCGCCTTCTCTT **(UPF3)**
TCACAGTCCGATCTCTTATCTCAAATTGATCCTCGTTTCGCTGATCGTTACAATTGGGTTTCGTTTCGTCCTGGG
AAGTCCAGgttagggtttatttctcgttgggtcttgaatttggaattttggtttctatgaattttagggttac
gaatttttgtaatttaagaagatgatggaaattaaagaagattgtatttgattatgttggaaattgaagattatt
gtatagattttgttacttagttgattgtagagaaattatacttagtagtattgggtttttttagtgatgtgtg
tgtgtgtttgtgtttttgtggtgtcttcagCTATAAAAATCAGAAGTATTCACGGGCCTATGTAAGTTTCAAGGC
ACCAGAAGATGTTTATGAGTTCGCTGCATTTTTCAACGGGCATGTGTTTGTTAATGAAAAGGgtatttgactttt
taatctgtctttgtctgatggttgcatgtggttttagtagtgtgtgtatttgaagatgtgatttttgtggtttct
tttggtttagGTGCTCAGTTTAAGGCTATAGTTGAATATGCACCTTCTCAGCGTGTTCCGAAACCGAGTGATAAG
AAAGATCCTCGTGAAGGGTCTATTAGTAAAGATCCTGATTATCTTGAGTTTCTTAAGGTGATTGCACAACCTGTT
GAGAATCTTCCTAGTGCTGAAATCCAGTTGGAAAGAAGAGAAGCTGAGCAGTCTGgttcgtgtttcgtatttgca
atcattttgatgcttcttttgtctgaagaggttggttattctctagtgaattaatcgaaattactaacacaacaa
ttatttttcttagGTGCTTCAAAAGCGGCTCCCATTGTTACACCTCTTATGGAATTCATACGTCAAAAACGTGCC
ACTGTGATGGGACCCCAGgtacattatgctcacagttttacggggttttcacttattctacctagttgaatatca
gaactttcctgggacggatgtctctctattttatttatttcaaaatggaacttctattgttgatctctggtttct
gttcaagcaacagGGTTTATCTGATATTCGAAGAGGAGGTAGAAGAACCAGAGTAGTCTCTGCAAACAAGCCGAG
TCCAAGGCCCTCGAAACGTAACTCTGAAAAGAAAAAGgtttgtctctcacttttttaggagtcaatagtcagtgtc
gtctgccattatactcatctgttgccattatattttttcattctagTATGTGGAAAAAGAAAGTTCAAAGAATGTG
CCCCGGAAGACTACAGCAGACGTCAGCAGCTCTAAGCCAGATTATCGTCAGTCAAATTCAAGTGGAAAGGAACTA
CCAGGAAATGAAACTGgtaaaaaatcaacagctgttgttattcctaactagctaattacatattttttaaaaggcc
acgtttaactctttgtctttttgctactcaattgcagCCGCTATCATTGATAGCTCTCCCCCTGGGATAGCATTG
ACTATGGATTCTGGGAAGAAAAAGATTTTGCTCCTGAGATCAAAAGACCGAGACAATCCTGATgtaagcttttttt
gttctttatgaaagttgtgcaggctgtttgattgtaaacagaattataagccatgtaaatttcttatttgttaat
ggtatttttagAACCCTCCACCACAACCGGAACAGCATATAGACACTAATCTTTCTAGAAACTCCACGGATTCAA
GACAAAACCAGAAGAGTGATGTTGGTGGGAGGTTGATCAAGGGAATACTTCTGAGAAATGACTCTCGACCGAGCC
AGTCTTCCACTTTTGTGCAGTCTGAGCAAAGAGTGGAACCCTCAGAAGCAGAAAACTACAAACGACCTTCTCGAC
CAGCCAACACTCGAGCAGgttataaaggaatacaatttaagtgaaaatgtttgcttcccaagttgggctgataat
tcaaaatagatggtccatgtgcagGGAAAGATTATCATACTTCTGGTACCATCAGTGAGAAGCAAGAGAGGCGTA
CAAGAAACAAGGATAGACCTGATCGTGTTATGTGGGCTCCTCGTCGTGATGGTAGTGAGGATCAACCACTATCTT
CAGCAGgttctgtttctgttgttgaccaataaataattagtttcattttcatggttgtgtttctcctccatgaca
tttgataggttcttacagGAAACAATGGAGAAGTGAAAGACAGGATGTTCTCTCAAAGATCGGGAGAAGTGGTGA
ACTCCTCTGGTGGTCACACTCTTGAGAATGgttagttcatctgaagttggagaatgcataatgtgtcttgtgtca
ttattatatgaataaataaaagtgacgttggttctgccaaaactttttgactctatccagGTTCTGCCAGACATT
CTAGTCGCCGTGTTGGAGGTCGCAATAGAAAAGAAGAGGTGGTGATTGGCGAGGGTAAAACCTCCCGGAGAGGAA
GTGGTGGTGGTCCCAGTTCACATGAGgcaagccaaaccaaagtcttttcttactacttttcctctgtcctctttt
aaccatgtatagaagattcatttttactgtgttgcagAAGCAAATGTGGATCCAAAAACCATCATCCGGTACT**TG**
Atatattctcttaacatatggtaagcttcagtcgcactactaccctctcttacttgaaatttgtcaacacagttt
tatgattgttgtgacctttcaggctaaatatatgagccattccactggattttgtccttcaatgggaatctatag
atctcaatcgaaatggggaagtaacattatcaacatcacttgggttttggagacttttggtccaacgttgtaagt
cgtagtattgcacataaaagaggccgttctcaaagttaggaatcagctcaagatttatgagaagtgagataagaa

aaactcgtttgctactgccttttgaaggccctagcggttcacggtacctgcatccccccttttggcttgtgcggt
gtcacagacaagtgagtgagaagtgagaacttactctcgagtctgagtaatgaaaataaagtcaaaagtgtatag
ggagcgggaagcacagtgtgtcgggttttattttgggttcctgagaggccttagaacttgattgcacgcttttaa
ccatataagagtttgattactttgaagttaatttcttgctaagtaatattttcttgtggaagttagtttattttt
tatatgattagtctatttgtacttatttttgatttcaccaacataaaagttatgctgaaagtgatttcacgttta
gaaagaccatattcttgcgattaggattaaagagggttacataaattaatcaaatggtcaaaaattaggaaatga
gaactcacttgtggaagagaagtcataaggataagagaccaatatatatttccccttggggtcgagtttttttggg
cctggcttgttagttgttacaatcgagtgtgatgcgtcccatttcggcggagactttgaatgtgtattattgacc
tttgattcctttgtccatcatttgttttgtgtttgcttaaaattaattttttggtcttccccagtcgactatatac
tctcttggtcacttgtaacgaagtaacaaaatgaaatacatgtcacatgtgatatatttatacacagactacaat
gaaatcaagtatgttgactaagaagagaagaaaatgaagtttgttgactaaagaaaggtcaaaatttttagaaat
tatcatcaaatcataaagatagtaaaataatgttaacagtatgaagtgtgaactcatatcttcgaagagattgag
acagagtttagaaaatctcttacaaatctagtttctacaacctcaactaacattgtccaaatctgtctctatcct
ctgttttatttttttcttccggtggtacaaaaattcacaatgaactgtatttatcacaattaatgaccgattgct AT1G33990
tcttcacctactctctctctctctctctctctttggaagtaaccaaacaagcctatatgctgctgatcccatc
taggggccaagcctctcttctttaaaaacttttgacttccattttcttgagaattcccctttctgggttcttgat

B

cagaatcagtaaaacctgacaagaccagaagtctctgcgaaacaaactcgtactttatcacctgcgacgcggatc
acctattagattgattatacaccgtacgatgtattattataatcttcatcctacggtaagcccaataacaagccc
atttcacaaactatcagagtaattatctgccgtccgatgccttaaaacttaatccgacggttgaaacatataatt
aaggcccattagaggcccatataggttaaaaagtccacgtagaccgaagattgatttgtctacgggtattttaat
tttttcttttcccagaggaaagggaagagaggaaacaaaaattagggcaaaattagcgtattgatgtgtttcact
SmaI and changing reading frame
tttcttcttcttcttcttcatcgtgttcgtgtttgatttgcgactttctccaaggaaaatttagggttttcg**<u>ATG</u>**
AAGGAACC**CCGGG**TTTGCAGAAGAAGAAGGTGGTTGTTCGTCACTTGCCGCCTTCTCTTTCACAGTCCGATCTC
TTATCTCAAATTGATCCTCGTTTCGCTGATCGTTACAATTGGGTTTCGTTTCGTCCTGGGAAGTCCAGgttaggg
ttttattttctcgttgggtcttgaatttggaattttggtttctatgaattttagggttacgaatttttgtaattt
aagaagatgatggaaattaaagaagattgtatttgattatgttggaaattgaagattattgtatagatttttgtt
acttagttgattgtagagaaattatacttagtagtattgggtttttttttagtgatgtgtgtgtgtgtttgtgttt
ttgtggtgetcttcagCTATACAAATCAGAAGTATTCACGGCC<u>ATG</u>G...

A to C change for eliminating a termination codon

NcoI + first ATG of GUS

Supplementary Figure 1 The *UPF3* gene and introduction of the first intron of *UPF3* into *GUS*. **(A)** The genomic region including the *UPF3* gene (AT1G33980). Parts of the upstream and downstream genes are also shown. The color annotations are: black - untranscribed regions, red - 5' or 3' UTRs, orange - coding sequence, and light blue - introns. The initiation and termination codons of *UPF3* are indicated by green, underlined letters. **(B)** Introduction of the first intron of *UPF3* into *GUS*. The first 8 nt downstream the initiation codon were not altered, since they affect translational efficiency. The sequence highlighted in grey was eliminated. Sequences highlighted in yellow were added (the purposes are indicated in the figure). Only the first four amino acids of the resultant GUS reporter are identical to UPF3. Following this point, the reading frame was altered.
